# Comparative Analysis of the Protective Effect of Naringenin on Cardiovascular Parameters of Normotensive and Hypertensive Rats Subjected to the Myocardial Infarction Model

**DOI:** 10.3390/ph17101324

**Published:** 2024-10-04

**Authors:** Anelize Dada, Rita de Cássia Vilhena da Silva, Mariana Zanovello, Jeniffer C. Moser, Sabrina L. D. Orengo, Martina O. Cavichiolo, Eleine R. Bidinha, Thaise Boeing, Valdir Cechinel-Filho, Priscila de Souza

**Affiliations:** Programa de Pós-Graduação em Ciências Farmacêuticas (PPGCF), Universidade do Vale do Itajaí (Univali), Itajai 88302-901, SC, Brazil

**Keywords:** acute myocardial infarction, flavonoids, isoproterenol, naringenin

## Abstract

**Background:** Cardiovascular diseases rank as the top global cause of mortality, particularly acute myocardial infarction (MI). MI arises from the blockage of a coronary artery, which disrupts blood flow and results in tissue death. Among therapeutic approaches, bioactives from medicinal plants emerge as promising for the development of new medicines. **Objectives:** This study explored the effects of naringenin (NAR 100 mg/kg), a flavonoid found in citrus fruits, in normotensive (NTR) and spontaneously hypertensive (SHR) rats, both subjected to isoproterenol (ISO 85 mg/kg)-induced MI. **Results:** Post-treatment assessments indicated that NAR reduced blood pressure and minimized clot formation, particularly notable in the SHR group, which helps mitigate damage related to hypertension and ISO exposure. Additionally, NAR effectively restored KCl-induced contractility in the aortas of both NTR and SHR groups. NAR treatment reduced reduced glutathione (GSH) and lipid hydroperoxides (LOOH) values and recovered the activity of the antioxidant enzymes catalase (CAT) and glutathione-s-transferase (GST) in NTR groups. Moreover, myocardial damage assessed through histological analyses was reduced in groups treated with NAR. **Conclusions:** The results highlight significant pathophysiological differences between the groups, suggesting that NAR has protective potential against ISO-induced cardiac damage, warranting further investigation into its protective effects and mechanisms.

## 1. Introduction

Cardiovascular diseases (CVD) represent the main causes of mortality in Brazil and on a global scale, according to a report by the Brazilian Society of Cardiology in 2019 [1]. Among ischemic diseases, acute myocardial infarction (MI) plays a crucial role as one of the most common medical emergencies, resulting from the acute obstruction of a coronary artery, followed by interruption of blood flow [2]. From a pathological point of view, MI is characterized as “myocardial cell death due to prolonged ischemia”. Symptoms associated with this condition can include discomfort in the upper limbs, chest pain, and even jaw pain, manifesting at rest and during physical activities [3]. The most critical therapeutic intervention in the treatment of MI is unblocking the affected artery. There are two main approaches to achieving this objective: mechanical unblocking, which involves the coronary angioplasty procedure, and the use of fibrinolytics, which consists of dissolving the clot using medications. The option for fibrinolytic therapy is reserved for situations in which angioplasty is not viable, as this approach can carry the risk of bleeding. Furthermore, the treatment of MI involves the use of other medications, which aim to prevent the formation of new clots, avoid arrhythmias, control cholesterol levels, and stimulate healing in the affected region [4]. Several MI models have been previously employed, including invasive procedures such as aortic banding and ligature of the aortic artery; however, these methods have been associated with a high risk of mortality. Currently, infarct models in rats are often established through the administration of doses of isoproterenol (ISO), which represents a less invasive approach. ISO, a β-adrenergic agonist, is capable of inducing changes similar to those observed in human MI [5]. Such changes include myocardial fibrosis, necrosis, inflammation, heart failure, and a reduction in antioxidant levels [6]. The possible underlying mechanism may be associated with oxidative stress, resulting from increased generation of reactive oxygen species and/or depletion of antioxidant defenses. The level of nitric oxide, regulated by the compromised signaling pathway of endothelial nitric oxide synthase (eNOS), plays a crucial role in myocardial damage [7].

Hypertension is recognized as one of the main risk factors for the development of other cardiovascular diseases, such as the MI [1]. Spontaneously hypertensive rats (SHR), an animal model developed through genetic crosses between isogenic strains, exhibit a remarkable similarity to the symptoms of hypertension in humans, thus becoming excellent models for studies related to hypertension. In addition to hypertension, SHR also develops cardiac hypertrophy and other systemic alterations, reflecting pathological aspects observed in patients with hypertension [8]. 

Crucial measures to prevent arterial obstruction and, therefore, MI, include regular physical activity, adopting a balanced diet, quitting smoking, and controlling risk factors such as diabetes, high blood pressure, and high cholesterol levels. At the same time, alternative approaches deserve to be highlighted, such as the use of medicinal plants, which have been employed as preventive treatments due to the pharmacological properties that different plant species can offer [9]. Plant-derived products demonstrate considerable potential for application as pharmaceuticals, with various important pharmacological activities of these substances already documented. Alkaloids, terpenes, and polyphenols represent the main classes of pharmacologically active phytochemicals that are synthesized by a wide range of plants. Many of these compounds have been employed in medicine and serve as starting points in the search for new drugs. Among them, flavonoids stand out as the most common group of polyphenolic components, offering significant potential and advantages for the prevention and treatment of cardiovascular diseases [7,8]. Exhibit beneficial properties for human health and can be obtained from fruits and vegetables, such as citrus fruits, which are represented for around 95% of the total flavonoids [9]. Research conducted in Finland, involving approximately 10,000 male and female individuals, investigated the relationship between flavonoid consumption and the risk of cardiovascular disease, revealing a 20% reduction in the risk of cerebrovascular disease among those who ingested higher levels of flavones daily [10].

Naringenin, a citrus flavanone, is a representative example of this class of compounds, which can be found in two forms: as naringin, in its glycosidic form, and as naringenin, the corresponding aglycone. Orhan et al. [11] demonstrated that naringenin contributed to reducing LDL and triglyceride levels, in addition to increasing HDL and strengthening antioxidant defenses. In models of myocardial ischemia-reperfusion, naringenin has been shown to be a cardioprotective agent [12]. However, despite the abundance of data on the biological effects of naringenin, few studies have explored its therapeutic properties in the context of myocardial infarction associated with systemic arterial hypertension (SAH). SAH is a highly prevalent chronic condition, affecting approximately one-third of the global population. It is considered one of the main risk factors for cardiovascular diseases, with additional implications such as atherosclerosis, MI, heart failure, stroke, and coronary and kidney disease [1]. Given the high rate of SAH in the population, along with the scarcity of studies that experimentally investigate the relationship between MI and hypertension, this study aimed to evaluate the therapeutic benefits of naringenin on cardiovascular parameters of normotensive (NTR) and spontaneously hypertensive (SHR) rats subjected to MI.

## 2. Results

### 2.1. Analysis of Baseline and after Treatment Arterial Pressure Values in NTR and SHR Groups

**Figure 1:**
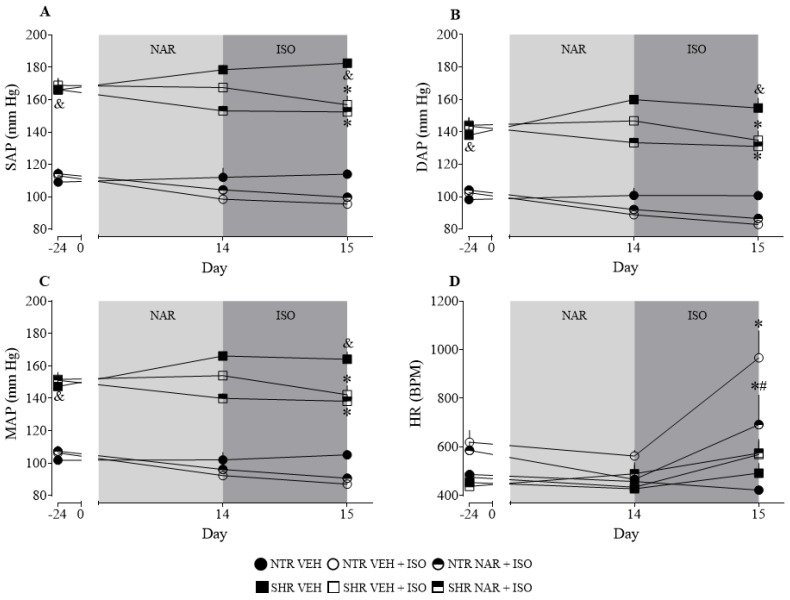
Differences between arterial pressure values and heart rate of the experimental groups via plethysmography (Tail-cuff). (**A**) Systolic arterial pressure (SAP); (**B**) Diastolic arterial pressure (DAP); (**C**) Mean arterial pressure (MAP); (**D**) Heart rate (HR). The results were expressed as the mean ± standard error of the mean (n = 6–8). Statistical analysis between groups was verified using two-way analysis of variance (ANOVA) followed by a Bonferroni post-test. * *p* < 0.05 when compared to the VEH. & *p* < 0.05 when compared to the NTR VEH group. # *p* < 0.05 when compared to the ISO group (85 mg/kg).

As observed in Figure 1, the SHR groups have elevated BP values compared to the NTR groups, validating the presence of hypertension. It is noted that after treatment with naringenin (NAR), there was a decrease in BP in the SHR groups and a significant prevention in the ISO-induced increase in HR (approximately 17% reduction).

### 2.2. Evaluation of Body Weight, Water Consumption, and Feed Intake in the NTR and SHR Groups

As observed in Table 1, no statistically significant differences were observed between the groups, except for the NTR group treated with NAR + ISO, where the p-value is close to the limit of statistical detection, suggesting a considerable weight loss. Although we cannot assert that the weight reduction observed in the NTR NAR + ISO group is directly attributed to the NAR treatment, there are studies in the literature that support this effect. Snoke et al. [13] demonstrated that groups treated with NAR showed a reduction in cumulative food intake and, consequently, a decrease in body weight. Similar results were obtained by Burke et al. [14] in studies with mice treated with NAR. Additionally, a study conducted with 8-week-old Wistar rats, subjected to a high-cholesterol diet and treated with NAR, showed that the dose of 100 mg/kg of NAR was able to reduce body weight compared to the group untreated with NAR [15].

In addition to body weight, the water consumption of the groups was monitored throughout the experiment. No significant changes were observed in any of the NTR groups, while a reduction in water consumption was observed in the SHR group treated with NAR + ISO. Similarly, the daily feed consumption of the groups was monitored, and no significant differences were found between the groups. However, when evaluating the consumption post-ISO induction, a 40% decrease in feed consumption was noted in the SHR group administered ISO and a 46% decrease in the SHR group treated with NAR + ISO, with no changes in the NTR groups.

### 2.3. Evaluation of the Relative Weight of the Heart, Aorta, Kidneys, and Liver

**Figure 2:**
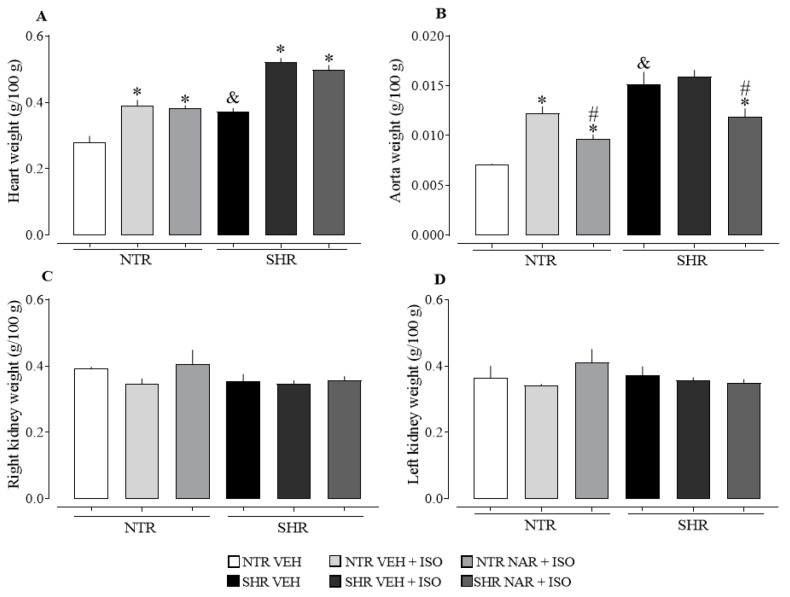
Weight of cardiac, aortic, and renal tissues obtained from the NTR and SHR groups. (**A**) heart weight; (**B**) aorta weight; (**C**) weight of the right kidney; (**D**) weight of the left kidney. The results were expressed as the mean ± standard error of the mean (n = 6–8). Statistical analysis between groups was verified using one-way analysis of variance (ANOVA) followed by a Bonferroni post-test. * *p* < 0.05 when compared to the VEH group of its respective group. # *p* < 0.05 when compared to the ISO group (85 mg/kg), of its respective group. & *p* < 0.05 when compared to the NTR VEH.

When evaluating Figure 2A, the SHR vehicle-treated group exhibits a significant increase in heart weight compared to the NTR vehicle group, indicating cardiac hypertrophy. However, after inducing infarction with ISO, there was an increase in heart weight in both NTR and SHR groups. Treatment with NAR for 14 days failed to reverse this cardiac alteration, suggesting that despite the significant reduction in blood pressure, a longer treatment period may be necessary to observe an improvement in this condition. In panel B, it is noted that the aorta of the SHR groups showed a significantly higher weight than that of the NTR groups. On the other hand, treatment with NAR demonstrated an ability to reduce the weight of the aorta compared to the corresponding vehicle and ISO-treated groups.

When evaluating kidney weight (panels 2C and D), no significant differences were observed between the groups. Liver weight evaluation did not reveal significant differences between the NTR and SHR groups, as well as among the treated groups.

### 2.4. Analysis of Clot Formation in Blood Samples from NTR and SHR Groups

**Figure 3:**
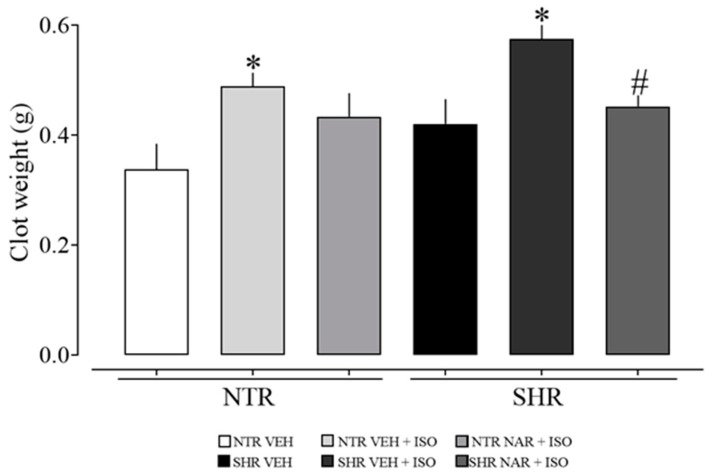
Clot formation in blood samples obtained from the NTR and SHR experimental groups. The results were expressed as the mean ± standard error of the mean (n = 6–8). Statistical analysis between groups was verified using one-way analysis of variance (ANOVA) followed by a Bonferroni post-test. * *p* < 0.05 when compared to the VEH group of its respective group. # *p* < 0.05 when compared to the respective ISO group (85 mg/kg).

Figure 3 shows that ISO significantly increased clot formation in both the NTR and SHR groups, but concurrently, NAR significantly reduced the weight of blood clots in the SHR groups. This suggests a reduction in risk factors associated with arterial thrombosis in hypertensive animals. However, these preliminary findings still require further detailed investigations to elucidate the effects of NAR on other blood parameters.

### 2.5. Evaluation of Blood Parameters in NTR and SHR Groups

As presented in Table 2, no statistically significant differences were observed in the levels of urea, creatinine, Na^+^, K^+^, and Cl^−^ between the NTR and SHR vehicle groups. However, a significant decrease in Ca^2+^ levels was identified in the SHR VEH group. When evaluating the groups administered with ISO, a decrease in these levels was noted in both groups, indicating impairment in Ca^2+^-mediated signaling pathways. Additionally, an increase in urea levels was observed in the ISO groups compared to their respective VEH groups. Notably, NAR demonstrated efficacy in preventing this increase in urea in the NTR group. A significant elevation in Na^+^ and K^+^ values was also observed in the SHR ISO group treated with NAR.

In addition to the analyses demonstrated above, blood glucose levels were also evaluated, which did not show significant differences between the groups.

### 2.6. Analysis of Cardiac Damage Markers NTR and SHR Groups

**Figure 4:**
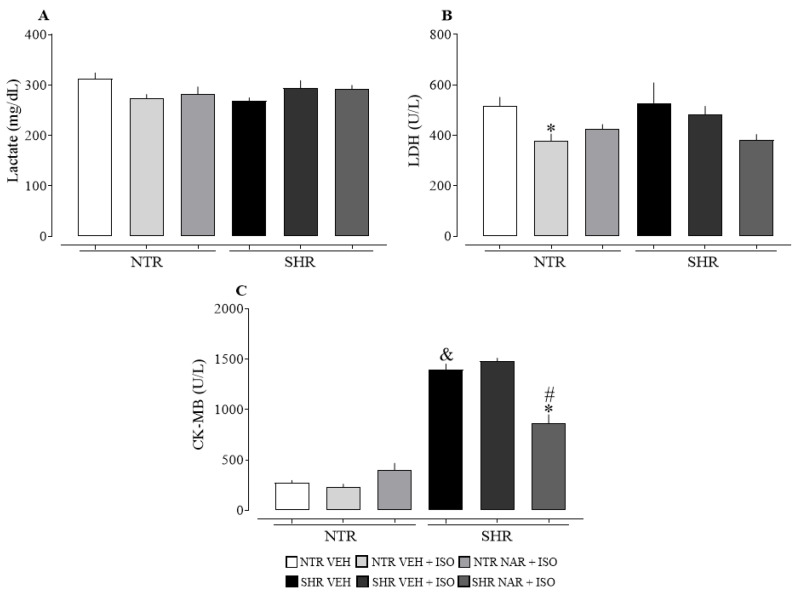
Plasma levels of lactate, LDH, and CK-MB in the NTR and SHR experimental groups. (**A**) lactate; in (**B**) LDH; in (**C**) CK-MB. The results were expressed as the mean ± standard error of the mean (n = 6–8). Statistical analysis between groups was verified using one-way analysis of variance (ANOVA) followed by a Bonferroni post-test. * *p* < 0.05 when compared to the VEH group of its respective group. # *p* < 0.05 when compared to the ISO group of its respective group. & *p* < 0.05 when compared to the NTR VEH.

Analyzing Figure 4, it is noted that lactate (panel A) did not show a significant difference between the groups. On the other hand, a reduction in LDH levels (panel B) was observed in the NTR VEH + ISO group. When evaluating CK-MB levels, it was observed that NAR had a positive impact on reducing these levels in the SHR groups, although it was not effective in the NTR groups. There is also a significant difference in values between the SHR and NTR groups, suggesting an extent of cardiac damage established by the condition of hypertension.

### 2.7. Evaluation of Aortic Responsiveness to Vasoconstrictors Obtained from NTR and SHR Groups

**Figure 5:**
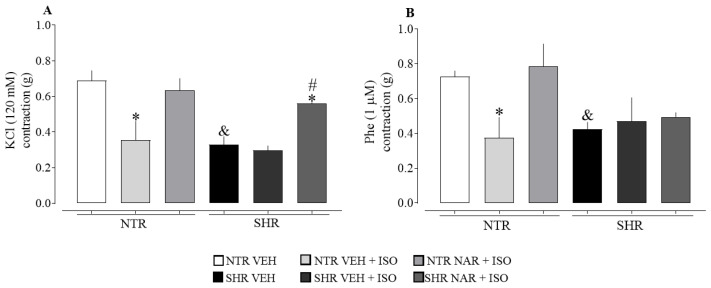
Contraction induced by KCl and phenylephrine (Phe) in isolated aortic rings from NTR and SHR groups. (**A**) KCl-induced contraction; (**B**) Phe-induced contraction. The results were expressed as the mean ± standard error of the mean (n = 6–8). Statistical analysis between groups was verified using one-way analysis of variance (ANOVA) followed by a Bonferroni post-test. * *p* < 0.05 when compared to the VEH group of its respective group. # *p* < 0.05 when compared to the ISO group (85 mg/kg) of its respective group. & *p* < 0.05 when compared to the NTR VEH.

Regarding the pathophysiological difference between the aortas obtained from NTR and SHR groups (i.e., treated only with VEH and not exposed to ISO), there is a compromised contractile response in SHR for both constrictors (KCl and Phe), as shown in Figure 5. On the other hand, ISO administration did not intensify the hypocontractility observed in SHR aortas. This differs from the response in NTR aortas, where a significant reduction in contractility was seen for both tested vasoconstrictors. Additionally, it was found that treatment with NAR was able to restore the contractility induced by KCl, suggesting a protective effect of NAR against ISO-induced aortic damage.

### 2.8. Analysis of Enzymatic and Non-Enzymatic Markers of Oxidative Stress

The results are expressed in Table 3, revealing a significant increase in LOOH levels in the groups exposed to ISO. On the other hand, the group treated with NAR exhibited a significant reduction in these markers compared to the respective VEH + ISO groups, suggesting the potential protective effect of NAR against lipid peroxidation. This effect may be attributed to its direct antioxidant action or its protective capacity against the damage induced by ISO administration. The SHR groups exhibit higher levels of GSH compared to the NTR groups. Additionally, there is a decrease in GSH levels in the groups treated with ISO, as ISO causes alterations in antioxidant processes, leading to oxidative stress. On the other hand, when evaluating the activity of the antioxidant enzyme superoxide dismutase (SOD), responsible for converting superoxide anion (O_2_^−^) into hydrogen peroxide (H_2_O_2_) [16], no significant differences were observed between the groups.

When evaluating the levels of catalase (CAT) activity, it is observed that the SHR groups have higher values than the NTR groups. However, the NTR groups exposed to ISO undergo a considerable increase in CAT and GST levels. On the other hand, in the SHR groups, there is a decrease in CAT and GST levels when exposed to ISO. Treatment with NAR was able to restore CAT and GST activity in the NTR groups, but not in the SHR groups.

### 2.9. Evaluation of Inflammatory Markers in the Cardiac Tissue of NTR and SHR Groups

In the analysis of Table 4, no significant differences were observed in myeloperoxidase (MPO) enzyme activity between the experimental groups, while N-acetyl-beta-D-glucosaminidase (NAG) showed elevated activity in the SHR VEH group compared to the NTR VEH group. Additionally, in the presence of ISO, the values increased significantly in the NTR groups. The levels of nitrite (NO_3_^−^) were higher in the SHR VEH group compared to the NTR VEH group, and ISO administration elevated NO_3_^−^ levels in both NTR and SHR groups. On the other hand, NAR significantly reduced these levels in SHR compared to the ISO-treated group.

### 2.10. Histological Analysis with Hematoxylin and Eosin Staining

**Figure 6:**
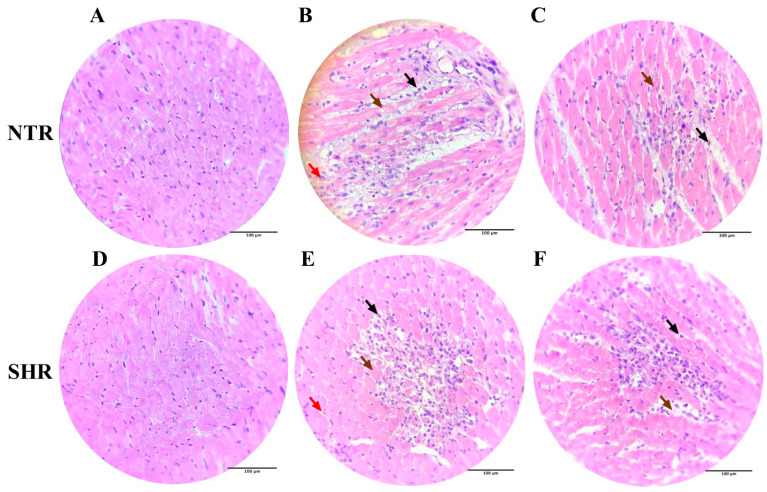
Histology of cardiac tissue on slides stained with Hematoxylin and Eosin (H and E 400×) in the different NTR and SHR experimental groups. (**A**) NTR VEH; (**B**) NTR VEH + ISO; (**C**) NTR NAR + ISO NTR; (**D**) SHR VEH; (**E**) SHR VEH + ISO; (**F**) SHR NAR + ISO. Black arrows indicate inflammatory cells (leukocytes) among the necrotic myocardiocytes. Red arrows indicate lipofuscin, a yellowish-brown pigment composed of highly oxidized proteins, lipids, and metals. Brown arrows indicate necrosis.

In Figure 6, inflammatory cells (leukocytes) were observed among necrotic myocardial cells, lipofuscin (brownish-yellow pigment composed of highly oxidized proteins, lipids, and metals), and necrosis. Additionally, a decrease in these markers is noted in the cardiac tissue of the groups treated with NAR.

### 2.11. Histological Analysis with Masson’s Trichrome Staining

**Figure 7:**
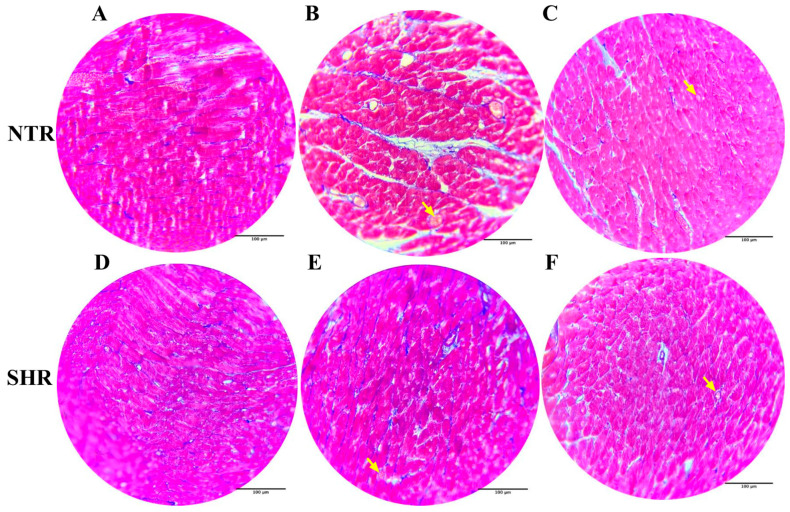
Histology of cardiac tissue on slides stained with Masson’s Trichrome (Blue stained-MT 400×) in the different experimental groups NTR and SHR. (**A**) NTR VEH; (**B**) NTR VEH + ISO; (**C**) NTR NAR + ISO; (**D**) SHR VEH; (**E**) SHR VEH + ISO; (**F**) SHR NAR + ISO. Yellow arrows indicate vascular congestion.

In Figure 7, the yellow arrows indicate vascular congestion in the groups exposed to ISO. Additionally, a lesser extent of damage is observed in the cardiac tissue of the groups treated with NAR.

### 2.12. Evaluation of Collagen Content in Myocardial Samples from NTR and SHR Groups

**Figure 8:**
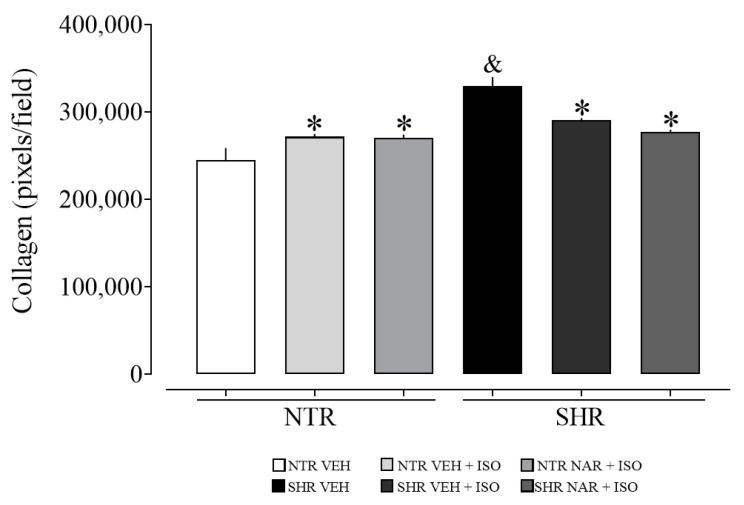
Collagen quantification was obtained from the NTR and SHR experimental groups. The results were expressed as the mean ± standard error of the mean (n = 6–8). Statistical analysis between groups was verified using one-way analysis of variance (ANOVA) followed by a Bonferroni post-test. * *p* < 0.05 when compared to the VEH group of its respective group. & *p* < 0.05 when compared to the NTR VEH.

Upon evaluating Figure 8, it is noticeable that the SHR VEH group exhibited a higher amount of collagen compared to the NTR VEH group. Additionally, it is suggested that ISO may induce cardiac hypertrophy in the NTR groups, as evidenced by the increase in collagen levels, which was not observed in the SHR groups, where there was a reduction in these levels.

## 3. Discussion

Given the high incidence of SAH and the scarcity of studies linking this comorbidity to the development of MI, the main objective of this study was to evaluate the protective effects of naringenin in an MI model. In addition, specific parameters were assessed to confirm cardiac damage and the protective effects of naringenin. With a large body of literature associating naringenin with the prevention of cardiac damage in normotensive animals (NTR), we hypothesized that it could also protect, and reverse conditions associated with hypertension and MI. Our findings demonstrated protective effects of naringenin against cardiac changes, restoring the activity of antioxidant enzymes, reducing clot formation, and recovering aortic contraction in SHR groups.

Naringenin has been extensively investigated due to its cardioprotective effects, with several studies highlighting its effects on cardiovascular diseases, especially acute myocardial infarction (MI). In a study conducted with mice subjected to isoprenaline to induce cardiac hypertrophy, groups treated with naringenin demonstrated positive effects in preventing hypertrophy [17]. Wang et al. [18] also observed a beneficial effect of naringenin in reducing atherosclerosis. Additionally, naringenin showed to be protective against myocardial ischemia/reperfusion injury [19]. However, to date, literature data confirming the protective effect of naringenin in MI models associated with comorbidities such as hypertension have not been found.

To confirm the presence of systemic arterial hypertension (SAH) in the SHR group, we initially measured blood pressure (BP) in the NTR and SHR groups before starting the treatments. This study’s findings are in line with those of Wei et al. [20], who demonstrated that systolic blood pressure (SBP) in SHR at 7 weeks is significantly higher than in NTR. Regarding heart rate (HR), the results corroborate previous findings by Dickhout et al. [5], which showed that SHR, from the sixth week of life, have elevated SBP values compared to NTR, but without significant differences in HR.

The data found in the evaluation of BP in the SHR groups corroborate with what was described by Liu et al. [15], where they demonstrated that NAR at doses of 50 and 100 mg/kg for 4 weeks was able to reduce SBP and diastolic blood pressure (DBP) in the SHR groups. The increase in HR in the NTR ISO group is directly related to the administration of ISO, a synthetic catecholamine acting as an agonist of β-adrenergic receptors, which, in response to its action, has a stimulating effect on the heart, both in HR and in contractile force [21]. It is noteworthy that SHR presents established cardiac hypertrophy due to high blood pressure levels (as will be discussed in the next paragraphs), which possibly prevents the cardiac cell response to ISO from being similar to the NTR group.

Cardiac hypertrophy, one of the most serious outcomes of SAH, is evidenced in this study, also seen in the findings of Jordão et al. [22]. Almeida et al. [23] corroborates this result by demonstrating that the SHR groups show a relative increase in cardiac weight compared to the NTR groups. The increase in heart weight in the ISO groups indicates an aggravation of cardiac hypertrophy, as expressed by Yin et al. [24], where the heart weight in the groups treated with ISO was higher than the control group. Khalil et al. [25] described a similar increase in cardiac weight in the NTR groups subjected to ISO-induced infarction compared to the untreated control groups. Ventricular hypertrophy is recognized as an adaptive mechanism of the cardiac muscle in response to pressure or volume overload. The literature indicates that this increase in ventricular mass is associated with an increase in cardiovascular morbidity and mortality in different populations, including the elderly and individuals with SAH [26,27,28].

Histological analyses of heart tissues were performed to confirm the damage caused by ISO and the protective potential of treatment with NAR, as well as to determine the collagen content in the heart tissue samples. Collagen, abundantly found in the myocardium [29], increases in quantity in states of hypertrophy [30]. Research indicates that cardiac hypertrophy arises from the remodeling of the extracellular matrix (ECM) and can be driven by conditions such as hypertension [31]. All this information is corroborated by the results of this study. Furthermore, our findings are similar to those reported by Patel et al. [32], who demonstrated the damage caused by ISO in histological analyses. Additionally, Shahzad et al. [33] also evidenced vascular congestion in groups administered with ISO in their study.

The higher weight of the aorta in SHR corroborates the findings of Jordão et al. [22], which indicate that the 8-week-old SHR groups exhibit more pronounced hypertrophy in the aortic wall and a larger total volume of the thoracic aorta compared to the NTR groups. Hypertension promotes changes in vascular structure, resulting in wall thickening, lumen reduction, hypoperfusion, and decreased oxygen transport to tissues [21]. Therefore, this effect of NAR on vascular damage resulting from hypertension can be considered beneficial. Hypertension is associated with vascular changes that manifest through endothelial dysfunction, increased vascular contractility, and arterial remodeling [34]. However, these alterations in vascular structure constitute a dynamic process in response to chronic hemodynamic conditions [35], which possibly explains the lower contractile capacity observed in the aortas of SHR. These findings corroborate the results of Gendron et al. [36], who evidenced a lower contractile response in SHR groups compared to NTR groups. For a better understanding of the results of this study, it is relevant to highlight that the activation of β-adrenergic receptors in blood vessels leads to relaxation [37], which could explain the reduced responsiveness observed in NTR. Considering the inherent changes in the hypertensive process in the SHR groups, exposure to ISO does not cause the same responses when compared to NTR, since basal responsiveness is already affected to a lesser extent.

In addition, in situations of vascular injury, blood coagulation is triggered to contain the bleeding. However, the formation of clots in inappropriate locations can lead to serious complications such as MI, stroke, and thrombosis [38]. Aissa et al. [39] associated hypertension with an increase in thrombi on the vascular wall. Although coagulation is a natural body process essential for healing and bleeding control, it can occur excessively and become deregulated under certain circumstances. A clot can completely obstruct blood flow in an artery, resulting in the partial or total death of the tissue it supplies, as in the case of compromised cardiac tissue due to coronary flow obstruction.

The enzyme CK-MB, predominantly found in cardiac muscle, plays an important role in detecting cardiac injuries, particularly during MI [40]. Additionally, LDH and lactate levels are evaluated; although these alone are not sufficient to diagnose cardiac damage, when combined with CK-MB, they can support the diagnosis of MI [41]. Therefore, the findings of this study show that SHR groups have greater cardiac damage caused by hypertension. Furthermore, NAR was effective in reversing these values, suggesting that it has a positive effect on reducing the damage caused by both ISO and hypertension. Shahzad et al. [33] observed significantly higher levels of LDH in groups administered with ISO (100 mg/kg), which contrasts with the findings of this study.

The administration of ISO triggers a series of systemic changes, including the generation of reactive species and/or depletion of antioxidant defenses. NAR is recognized for its antioxidant efficacy [42], and to validate this, markers of oxidative stress were evaluated. Lipid hydroperoxide (LOOH) levels were assessed since lipid peroxidation can result in cellular alterations and tissue damage, serving as an indirect indicator of oxidative stress in tissues [43]. In the present study, an increase in LOOH levels was observed in the groups administered ISO, however, NAR was able to reverse these changes, providing a possible protective effect against oxidative damage. These results corroborate with what was expressed by Patel et al. [32] and Yin et al. [24], where LOOH values increased in NTR groups subjected to ISO administration. Reduced glutathione (GSH) plays an essential role in vital biological processes, acting in cellular defense against oxidative stress and in the elimination of foreign substances from the body [44]. Yuan et al. [45] also observed higher GSH values in the hearts of SHR groups, although without statistically significant differences compared to NTR. Shahzad et al. [33] also found a reduction in GSH levels in NTR groups treated with ISO compared to the vehicle groups. Both studies corroborate what was found in this research, SHR groups demonstrated higher GSH values. Catalase (CAT) is an essential enzyme in the detoxification process of reactive oxygen species, converting H_2_O_2_ into water (H_2_O) and oxygen (O_2_) [46]. On the other hand, glutathione S-transferase (GST) is responsible for the conjugation of xenobiotics with glutathione (GSH), reducing their toxicity [44]. Álvarez et al. [47] found a significant increase in CAT levels in SHR groups compared to NTR, corroborating the data found here. Contrary to what was found in this study, Yin et al. [24] observed a reduction in CAT and GST levels in NTR groups treated with ISO.

Myeloperoxidase (MPO), a natural component of neutrophils, is associated with inflammatory processes caused by the accumulation of neutrophils [48,49]. On the other hand, NAG is considered a marker of macrophage infiltration and, therefore, of inflammation [50]. Nitrite (NO_3_^−^), an indirect marker of nitric oxide (NO) production, has a longer half-life than NO and is generally associated with oxidative stress [51,52,53,54]. Thus, our study suggests the presence of chronic inflammation in SHR, and that ISO contributes to the development of inflammation in NTR, since there was a significant increase in nitrite values in SHR groups and in groups that received ISO administration. On the other hand, NAR prevented this increase in SHR groups, suggesting a protective effect against damage caused by ISO.

**Figure 9:**
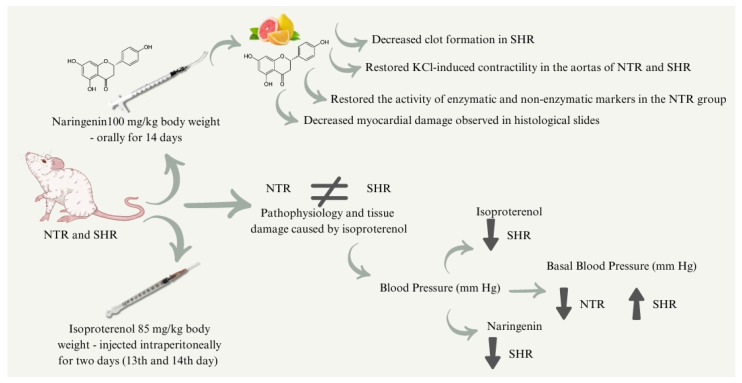
Final diagram. Summary of the study findings and effects of naringenin. Arrows pointing upwards denote an increase, while arrows pointing downwards denote a decrease.

Finally, in this study, protective activities of naringenin (NAR) were observed in NTR, as previously found in other studies. In addition, protective effects of NAR were observed in spontaneously hypertensive rat (SHR) groups, suggesting a promising effect against cardiac changes caused by preexisting hypertension and ISO administration. It is suggested that the mechanisms by which NAR protects against cardiac changes are associated with the recovery of antioxidant enzyme activity, reduction of blood pressure (in SHR), decreased clot formation, recovery of aortic contraction, and reduced tissue damage. The final diagram is summarized in Figure 9. However, despite these promising results, further studies with longer treatment durations are recommended, as well as exploring other pharmaceutical forms of naringenin. Additionally, studies should focus on more realistic models of MI pathophysiology to get closer to the pathophysiology that affects humans.

## 4. Materials and Methods

### 4.1. Drugs

The drugs used in this study for treatment (naringenin, with a purity of 95%) and induction of myocardial infarction (isoproterenol) were obtained commercially (Sigma Aldrich, San Luis, MI, USA) and prepared according to the manufacturer’s instructions. The dose of naringenin (100 mg/kg) was determined according to Liu et al. [15], where they demonstrated that NAR in doses of 50 and 100 mg/kg for 4 weeks was able to reduce SBP and DBP in the SHR groups. The dose of isoproterenol (85 mg/kg) was defined according to Khan et al. [55], who promoted the induction of infarction by ISO in NTR groups with a dose of 85 mg/kg.

### 4.2. Animals

Normotensive (NTR) and spontaneously hypertensive (SHR) Wistar rats aged 3 to 4 months were used, provided by the UNIVALI Vivarium. The animals were maintained at controlled room temperature (22 ± 2 °C), 12-h light/dark cycle, with free access to water and food. All methodologies and procedures proposed here were approved by the UNIVALI Animal Experimentation Ethics Committee (No. 017/22 and 005/23) and were conducted under all established ethical standards.

### 4.3. Blood Pressure Measurements via Plethysmography (Tail-Cuff)

The measurement of systolic blood pressure (SBP), diastolic blood pressure (DBP), mean arterial pressure (MAP), and heart rate (HR) was performed by plethysmography (Serial number: 007006; Bonther, Ribeirão Preto, SP, BR). To reduce the stress of the restraint required to measure blood pressure, the vehicle, and treated groups were trained to adapt the equipment in the week before the first blood pressure measurement. After adaptation in a heated room at 28–30 °C, the animals were placed in acrylic containment tubes and placed on the heated plate. The previously calibrated transducer was connected to a sphygmomanometer (placed on the animal’s tail) equipped with an automated inflation system, which is coupled to a data capture and conversion system connected to a computer and specific data acquisition software (Tail Plethysmography.Ink, Bonther, Ribeirão Preto, SP, Brazil).

### 4.4. Induction of Infarction by Isoproterenol

The animals were randomly divided into the following groups, each containing 6 to 8 rats: (I) VEH: vehicle group NTR; (II) VEH + ISO: vehicle + isoproterenol 85 mg/kg NTR; (III) NAR + ISO: naringenin 100 mg/kg + isoproterenol 85 mg/kg NTR; (IV) VEH: vehicle group SHR; (V) VEH + ISO: vehicle + isoproterenol 85 mg/kg SHR; (VI) NAR + ISO: naringenin 100 mg/kg + isoproterenol 85 mg/kg SHR.

Rats from Groups II, III, V, and VI were injected intraperitoneally with ISO (85 mg/kg body weight) over two days at 24-h intervals (13th and 14th day) to induce myocardial infarction; rats in Groups III and VI (treatment groups) were pre-administered orally and co-administered with naringenin (NAR; 100 mg/kg body weight) daily for 14 days and injected intraperitoneally with isoproterenol (ISO; 85 mg/kg body weight) for two days (13th and 14th day) at 24-h intervals to induce myocardial infarction. Twenty-four hours after the second dose of ISO injection (i.e., on day 15), rats were anesthetized using an intraperitoneal injection of xylazine and ketamine (10 and 80 mg/kg body weight, respectively). Blood was collected in tubes and centrifuged to separate the plasma, part of which was used for the clot formation model. The heart, liver, kidneys, and aorta were immediately excised and washed in ice-cold saline. For biochemical estimations, cardiac tissues were weighed and then homogenized in appropriate buffers. For histological evaluation, the apical ventricular portion of the hearts from all groups was collected and fixed in ALFAC solution.

### 4.5. Evaluation of Clot Formation

To evaluate clot formation, one mL of blood from each animal was added to the identified tube and placed in a water bath at 37 °C for 1 h. Afterwards, all clots formed were weighed, and the results were expressed in g.

### 4.6. Blood Tests

Na^+^ and K^+^ concentrations were measured using a flame photometer (model BFC-300; Benfer, São Paulo, Brazil). The instrument was calibrated with a standard solution containing different concentrations of Na^+^ and K^+^, and samples were read, previously diluted in distilled water (1:1000), under specific wavelengths. The contents of urea, creatinine, Cl^−^, Ca^2+^, lactate, lactate dehydrogenase (LDH), and creatine phosphokinase MB (CK-MB) were evaluated by colorimetric test following the manufacturer’s instructions (Bioclin, Belo Horizonte, MG, Brazil).

### 4.7. Evaluation of the Relative Weight of the Aorta, Kidney, Liver, and Heart

The relative weight of the thoracic aorta (with complete removal of the connective tissue), kidney (excluding the adrenal gland and adjacent adipose tissue), liver, and heart (after complete separation of the atria and base vessels) were determined using an analytical balance. The results were expressed as tissue weight per 100 g of animal weight.

### 4.8. Evaluation of Weight Gain, Food, and Water Consumption

The animals were weighed using a digital scale with an accuracy of 1 g. The animals were weighed every two days and on the last day of treatment for 15 days, during which food consumption was also evaluated (by weighing the food) and water consumption (measured with the aid of graduated cylinders) from both groups.

### 4.9. Evaluation of Vascular Reactivity in an Isolated Aorta Model

For the isolated aorta protocol, we used the same groups described above. After removal and weighing, the aorta was dissected from adipose tissue and adherent connective tissue and sectioned into 3–5 mm rings. Immediately after removal, isolated aortic rings, with functional endothelium, were maintained in organ baths containing 2 mL of PSS (PSS; pH 7.4; composition in mM: NaCl 115.3, KCl 4.9, CaCl_2_-2H_2_O 1.46, KH_2_PO_4_ 1.2, MgSO_4_ 1.2, d-glucose 11.1, NaHCO_3_ 25) under resting tension of 1g, maintained at 37 °C and continuously aerated with 95% O_2_ and 5% CO_2_. An interval of 60 min was respected for stabilization at the beginning of the experimental protocol as well as between each series of drug exposures. After the stabilization time, the rings were exposed to the vasoconstrictive substances KCl 60 mM and Phenylephrine (Phe) 1 µM. Contractions were expressed in grams (g). The recordings were obtained using isometric transducers, coupled to DATAQ Instruments data acquisition hardware connected to a computer with specific software integration, a signal amplifier, and connected to a computer containing specific integration software (WinDaq software, DATAQ Instruments, Akron, OH, USA).

### 4.10. Determination of Oxidative and Inflammatory Parameters

To analyze the enzymatic and non-enzymatic parameters of oxidative stress, heart tissue samples from the vehicle and treated groups were used. Cardiac tissue samples were homogenized in 200 mM phosphate buffer (pH 6.5, 4 °C) and centrifuged at 9000 RPM for 20 min at 4 °C. All experiments were conducted in duplicates. The homogenate was used to measure lipid hydroperoxide (LOOH) levels through adaptations of what was proposed by Jiang et al. [56] and reduced glutathione (GSH) as adapted from the protocol by Sedlak et al. [57]. After the homogenate was centrifuged at 9000 RPM for 20 min, the supernatant was used to evaluate glutathione *S*-transferase (GST) activity with adaptations to the model by Habig et al. [58], superoxide dismutase (SOD) adapted from the model proposed by Marklund and Marklund [59], and catalase (CAT) according to Aebi [60], with adaptations. The precipitate was used to measure myeloperoxidase (MPO) activity described by Bradley et al. [48] and adapted from Young et al. [61]. Protein concentrations were determined in all samples using Bradford reagent and bovine albumin as standard, following the manufacturer’s instructions.

Furthermore, the pellet supernatant was also used to determine N-acetyl-beta-D glucosaminidase (NAG) activity, following the method described by Bailey [62] with some adaptations. Nitrite levels, a marker of nitric oxide production, were quantified according to the method applied by Tsikas [63], with the necessary adaptations for the sample used.

### 4.11. Histological Analysis and Collagen Quantification

For histological evaluation, part of the cardiac tissue (apical ventricular portion) obtained from all groups was fixed in ALFAC solution (85% alcohol at 80%, 10% formaldehyde, and 5% glacial acetic acid) and subsequently dehydrated in 70% alcohol, embedded in paraffin, sectioned into 5 μm sections with the aid of a microtome, and stained with hematoxylin-eosin and Masson’s trichrome. The sections were viewed and photographed using a Basic Binocular Achromatic Microscope–K55-BA (Proway Optics and Electronics Co., Ltd., Ningbo, China) to characterize the histological changes. Collagen quantification was performed using ImageJ software version 1.54k (RRID: SCR_003070). Eight fields of the myocardium of each animal were photographed and quantified.

### 4.12. Statistical Analysis

The results were expressed as the mean ± standard error of the mean (n = 6 to 8 animals in each group). For statistical analysis, one- or two-way analysis of variance (ANOVA) was used, followed by the Bonferroni test, using GraphPad Prism version 8.0.1 for Windows (GraphPad Software, La Jolla, CA, USA). A *p* value less than 0.05 was considered statistically significant.

## 5. Conclusions

Taken together, the results of this study underscore a significant pathophysiological distinction between the NTR and SHR groups, evidenced by differences in blood pressure values and responses to ISO-induced tissue damage. The SHR groups exhibited greater resilience, likely due to pre-existing cardiac adaptations associated with SAH. Treatment with NAR revealed antihypertensive effects and a reduction in clot formation, particularly notable in the SHR group, which helps mitigate damage related to SAH and ISO exposure. Additionally, NAR effectively restored KCl-induced contractility in the aortas of both NTR and SHR groups and displayed antioxidant properties by rejuvenating the activity of enzymatic and non-enzymatic markers in the NTR group. It also demonstrated a significant reduction in nitrite levels in the SHR group compared to the ISO group. Moreover, myocardial damage, assessed through histological analyses, was reduced in groups treated with NAR. Concerning collagen quantification, which literature has previously elucidated, levels were elevated in SHR. Despite the relevant results obtained in the present study, further studies are required to deepen our understanding of the physiological alterations induced by ISO in the context of SAH and to fully clarify the protective effects of NAR.

## Figures and Tables

**Table 1:** Body weight (g) of the different experimental groups NTR and SHR.

Groups	Weight (g) Day 0	Weight (g) Day 15
NTR VEH	337 ± 21	340 ± 25
NTR VEH + ISO	340 ± 25	298 ± 19
NTR NAR + ISO	336 ± 14	287 ± 18 (*p* = 0.0547)
SHR VEH	291 ± 7	319 ± 15
SHR VEH + ISO	293 ± 7	291 ± 5
SHR NAR + ISO	291 ± 7	280 ± 6

VEH: vehicle group; VEH + ISO: vehicle + isoproterenol; NAR + ISO: naringenin + isoproterenol. The results were expressed as the mean ± standard error of the mean (n = 6–8). Statistical analysis between groups was verified using a two-way analysis of variance (ANOVA) followed by a Bonferroni post-test.

**Table 2:** Blood parameters in samples from the NTR and SHR groups.

Groups	Urea (mg/dL)	Creatinine (mg/dL)	Na^+^ (mmol/L)	K^+^ (mmol/L)	Cl^−^ (mmol/L)	Ca^2+^ (mg/dL)
NTR VEH	47.1 ± 1.3	0.5 ± 0.1	164.0 ± 11.5	21.2 ± 2.1	230.6 ± 9.2	9.6 ± 1.4
NTR VEH + ISO	85.8 ± 6.8 *	0.1 ± 0.01 *	138.0 ± 3.8	15.6 ± 2.5	209.2 ± 2.2	5.5 ± 0.1 *
NTR NAR + ISO	59.8 ± 7.0 #	0.2 ± 0.06	133.3 ± 5.1	15.4 ± 2.0	231.1 ± 7.2	6.0 ± 0.3 *
SHR VEH	51.8 ± 3.9	0.3 ± 0.03	163.2 ± 6.0	17 ± 1.7	207.1 ± 1.9	5.8 ± 0.4 &
SHR VEH + ISO	86.4 ± 3.4 *	0.2 ± 0.03	173.0 ± 6.3	15.6 ± 1.6	205.2 ± 2.1	5.5 ± 0.2
SHR NAR + ISO	81.0 ± 2.3 *	0.3 ± 0.06	192.0 ± 5.3 *	24.1 ± 2.8 #	211.1 ± 4.2	5.7 ± 0.1

VEH: vehicle group; VEH + ISO: vehicle + isoproterenol; NAR + ISO: naringenin + isoproterenol. The results were expressed as the mean ± standard error of the mean (n = 6–8). Statistical analysis between groups was verified using one-way analysis of variance (ANOVA) followed by a Bonferroni post-test. * *p* < 0.05 when compared to the VEH. # *p* < 0.05 when compared to the ISO group (85 mg/kg). & *p* < 0.05 when compared to the NTR VEH.

**Table 3:** Enzymatic and non-enzymatic indicators of oxidative stress.

Groups	LOOH (μmol/g of Tissue)	GSH (μg/g of Tissue)	SOD (U/ mg Protein)	CAT (μmol/mg/min)	GST (μmol/mg Protein/min)
NTR VEH	1.27 ± 0.10	868.5 ± 79.9	0.16 ± 0.02	0.09 ± 0.003	0.005 ± 0.003
NTR VEH + ISO	1.59 ± 0.14	794.1 ± 49.8	0.11 ± 0.006	1.03 ± 0.31 *	0.018 ± 0.003 *
NTR NAR + ISO	0.99 ± 0.01 #	824.7 ± 24.3	0.13 ± 0.01	0.16 ± 0.03 #	0.008 ± 0.001 #
SHR VEH	1.53 ± 0.16	1416 ± 109.1 &	0.17 ± 0.01	0.36 ± 0.04 &	0.015 ± 0.003
SHR VEH + ISO	2.16 ± 0.18 *	1299 ± 26.3	0.17 ± 0.008	0.20 ± 0.04 *	0.005 ± 0.001 *
SHR NAR + ISO	1.60 ± 0.08 #	1312 ± 80.2	0.17 ± 0.009	0.21 ± 0.02 *	0.004 ± 0.0008 *

VEH: vehicle group; VEH + ISO: vehicle + isoproterenol; NAR + ISO: naringenin + isoproterenol. The results were expressed as the mean ± standard error of the mean (n = 6–8). Statistical analysis between groups was verified using one-way analysis of variance (ANOVA) followed by a Bonferroni post-test. * *p* < 0.05 when compared to the VEH. # *p* < 0.05 when compared to the ISO group (85 mg/kg). & *p* < 0.05 when compared to the NTR VEH.

**Table 4:** Inflammatory markers in the cardiac tissue of NTR and SHR groups.

Groups	MPO (mD.O/μg Protein)	NAG (mD.O/μg Protein)	Nitrite (μM)
NTR VEH	3 ± 0.2	15.58 ± 1.38	0.48 ± 0.18
NTR VEH + ISO	3 ± 0.2	25.09 ± 1.65 *	5.01 ± 1.54 *
NTR NAR + ISO	4 ± 0.1	21.09 ± 0.82 *	3.21 ± 0.87
SHR VEH	4 ± 0.2	36.59 ± 2.34 &	5.47 ± 1.36 &
SHR VEH + ISO	4 ± 0.2	41.68 ± 2.24	10.94 ± 2.04 *
SHR NAR + ISO	4 ± 0.1	37.94 ± 3.17	6.32 ± 0.61 #

VEH: vehicle group; VEH + ISO: vehicle + isoproterenol; NAR + ISO: naringenin + isoproterenol. The results were expressed as the mean ± standard error of the mean (n = 6–8). Statistical analysis between groups was verified using one-way analysis of variance (ANOVA) followed by a Bonferroni post-test. * *p* < 0.05 when compared to the vehicle. # *p* < 0.05 when compared to the ISO group (85 mg/kg). & *p* < 0.05 when compared to the NTR vehicle.

## Data Availability

The raw data supporting the conclusions of this article will be made available by the authors upon request.
